# SARS-CoV-2 Omicron Variant Binds to Human Cells More Strongly than the Wild Type: Evidence from Molecular Dynamics Simulation

**DOI:** 10.1021/acs.jpcb.2c01048

**Published:** 2022-06-20

**Authors:** Hoang Linh Nguyen, Nguyen Quoc Thai, Phuong H. Nguyen, Mai Suan Li

**Affiliations:** † Life Science Lab, Institute for Computational Science and Technology, Quang Trung Software City, Tan Chanh Hiep Ward, District 12, Ho Chi Minh City 700000, Vietnam; ‡ Ho Chi Minh City University of Technology (HCMUT), Ho Chi Minh City 700000, Vietnam; § Vietnam National University, Ho Chi Minh City 700000, Vietnam; ∥ Dong Thap University, 783 Pham Huu Lau Street, Ward 6, Cao Lanh City, Dong Thap 8100, Vietnam; ⊥ CNRS, Universit́e de Paris, UPR9080, Laboratoire de Biochimie Th́eorique, Paris, France; Institut de Biologie Physico-Chimique, FondationEdmond de Rothschild, PSL Research University, Paris 75006, France; # Institute of Physics, Polish Academy of Sciences, al. Lotnikow 32/46, Warsaw 02-668, Poland

## Abstract

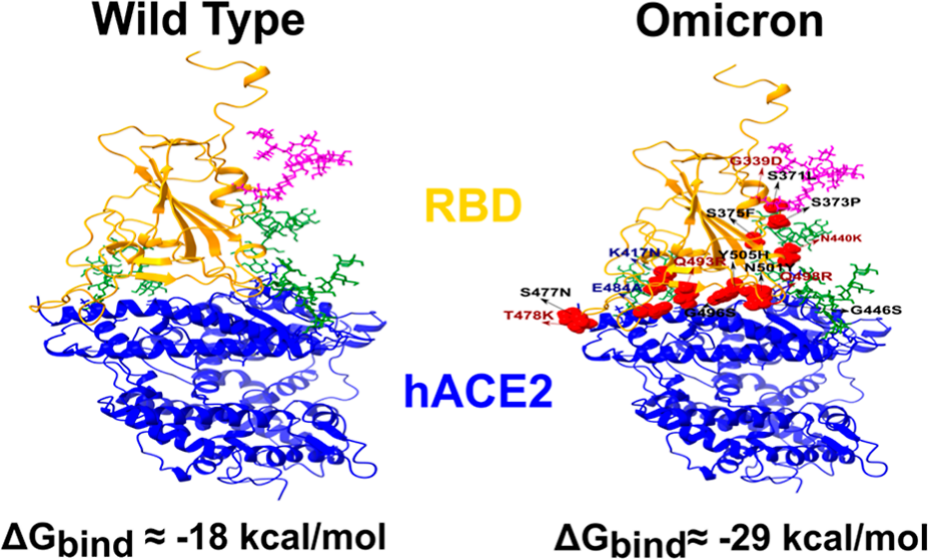

The emergence of the variant of concern Omicron (B.1.1.529) of the severe acute respiratory syndrome coronavirus 2 has aggravated the Covid-19 pandemic due to its very contagious ability. The high infection rate may be due to the high binding affinity of Omicron to human cells, but both experimental and computational studies have yielded conflicting results on this issue. Some studies have shown that the Omicron variant binds to human angiotensin-converting enzyme 2 (hACE2) more strongly than the wild type (WT), but other studies have reported comparable binding affinities. To shed light on this open problem, in this work, we calculated the binding free energy of the receptor binding domain (RBD) of the WT and Omicron spike protein to hACE2 using all-atom molecular dynamics simulation and the molecular mechanics Poisson–Boltzmann surface area method. We showed that Omicron binds to human cells more strongly than the WT due to increased RBD charge, which enhances electrostatic interaction with negatively charged hACE2. N440K, T478K, E484A, Q493R, and Q498R mutations in the RBD have been found to play a critical role in the stability of the RBD-hACE2 complex. The effect of homogeneous and heterogeneous models of glycans coating the viral RBD and the peptidyl domain of hACE2 was examined. Although the total binding free energy is not sensitive to the glycan model, the distribution of per-residue interaction energies depends on it. In addition, glycans have a little effect on the binding affinity of the WT RBD to hACE2.

## Introduction

The coronavirus disease (COVID-19) pandemic caused by severe acute respiratory syndrome coronavirus 2 (SARS-CoV-2)^1^ has killed more than 5.5 million people out of over 313 million confirmed cases worldwide.^2^ SARS-CoV-2 is a new member of the beta genera of genus Coronavirus.^1^ The virion comprises a single positive-strand RNA enveloped by a lipid bilayer with sphere-like shape. Among various virial proteins, the so-called spike (S) protein protruding from the lipid bilayer plays an important role in the invasion of host cells and in antibody binding.^3−7^ Binding of the receptor binding domain (RBD) of the S protein to the human angiotensin-converting enzyme 2 (hACE2) protein initiates entry of the virus to the host cell.^8,9^ Therefore, over the past two years, a lot of experimental^10,11^ and computational^12−15^ studies on the RBD-hACE2 interaction have been performed.

In November 2021, a newly emerging variant called Omicron (B.1.1.529) was reported as a variant of concern.^16^ This variant encodes a large number of genomic mutations including 32 mutations in the S protein^17^ (Table 1). The outbreak of the Delta variant has unleashed a devastating wave of the pandemic,^18−20^ but Omicron makes the virus spread even faster, bringing a lot of attention to its dominant role.^21^ Therefore, understanding the molecular mechanism underlying the interaction of Omicron with hACE2 is very important as it may shed light on the high transmissibility of this variant. A large number of mutations in the S protein are expected to drastically change this interaction, but different groups have reported conflicting experimental results. The binding affinity of the Omicron variant S protein to hACE2 was found to be higher (lower the dissociation constant *K*_D_) than that of the SARS-CoV-2 wild-type (WT) analogue^22,23^ (Table 2). However, Wu *et al.* reported that this binding affinity is comparable to that of the WT.^24^ Chan *et al.*(25) obtained *K*_D_ = 22 nM, which suggests that WT and Omicron have almost the same binding affinity if this value is compared to *K*_D_ of Omicron from Cameroni *et al.*(22) and Wu *et al.*(24) (Table 2). However, when this *K*_D_ value is compared with that of Zhang *et al.*,^23^ Omicron binds more closely to hACE2.

**Table 1:** Mutations in the S Protein of the Omicron Variant^a^

variant	mutations
Omicron	A67V, Δ69-70, T95I, G142D, Δ143-145, N211I, L212V, ins213-214RE, V215P, R216E, **G339D, S371L, S373P, S375F, K417N, N440K, G446S, S477N, T478K, E484A, Q493R, G496S, Q498R, N501Y, Y505H**, T547K, D614G, H655Y, N679K, P681H, N764K, D796Y, N856K, Q954H, N969K, L981F

a Residues located in the RBD are in bold. Δ indicates deletion.

**Table 2:** Dissociation Constant *K*_D_ of Complexes of SARS-CoV-2 Variants and hACE2^a^

variant	*K*_Dx_ (nM)
Wildtype	60.0 ± 1.4,^22^ 13.20,^23^ 16.6 ± 8.4,^18^ 22.0^25^
Omicron	25.3 ± 1.2,^22^ 8.85,^23^ 270.27 ± 3403.94^18^

a References to experimental studies are given.

Computational studies have also produced conflicting results. Using an artificial intelligence model and docking simulation, it was shown that Omicron is more contagious than the WT virus.^26,27^ Omotuyi *et al.* performed molecular dynamics (MD) simulations showing that Omicron exhibits a stronger interaction with hACE2,^28^ but binding free energy has not been reported. Kim *et al.* reported that Omicron RBD has a higher binding affinity to ACE2 compared to the WT using steered molecular dynamics (SMD) simulation and experimental microscale thermophoresis.^29^ However, the comparable binding affinity was obtained^18^ combining MD modeling with the molecular mechanics Poisson–Boltzmann surface area (MM-PBSA) method.

Since both experimental and computational results are inconsistent with each other, here we attempted to calculate the binding free energy of the WT and the Omicron variant of SARS-CoV-2 interacting with hACE2 using the MM-PBSA method. In contrast to previous studies,^18^ our all-atom model includes glycans flanked around the RBD of S protein. We have shown that the Omicron RBD binds to the peptidyl domain (PD) of hACE2 more strongly than the WT.

In both implemented glycan models, electrostatic interaction prevails over the van der Waals (vdW) interaction in binding of the WT and Omicron to human cells. In addition, Omicron displays a stronger electrostatic interaction with hACE2 than the WT.

We have identified three interface regions between RBD and hACE2 PD. The mutations help Omicron to improve interaction with hACE2 compared to the WT in two interface regions, while in another region, Omicron has weaker interaction with hACE2, suggesting that SARS-CoV-2 still has room for improving binding affinity with human cells.

Finally, our MD simulation without glycans showed that glycans have an insignificant effect on the binding free energy of RBD WT to hACE2 PD.

## Materials and Methods

### Molecular Dynamics Simulation

**Figure 1:**
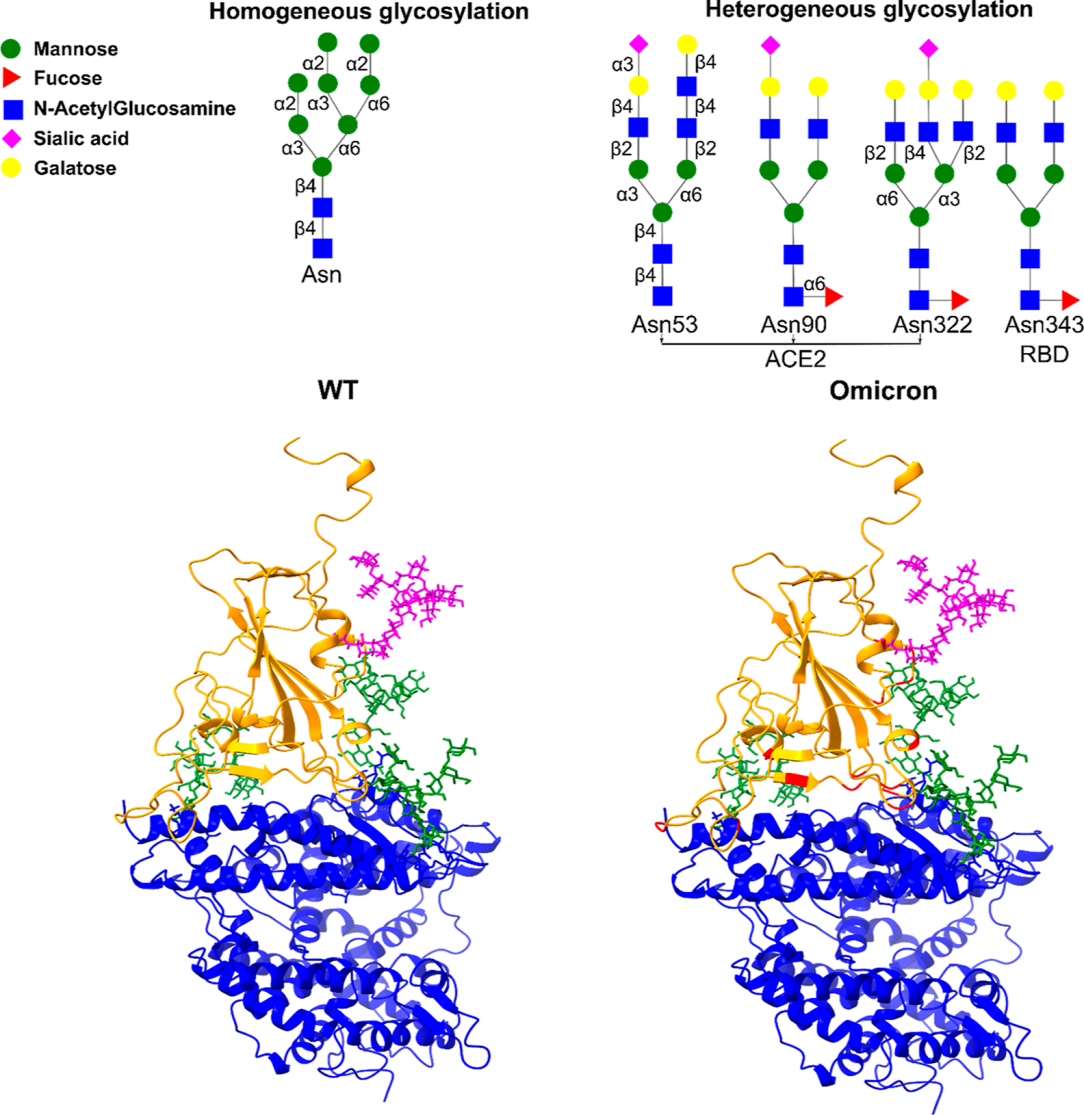
(Upper) Glycan models used in this work. Magenta refers to the glycan flanking RBD, while green refers to the three glycans surrounding hACE2. (Bottom) PDB structure of the WT RBD-hACE2 PD and Omicron RBD-hACE2 PD complexes. RBD is highlighted in orange, hACE2 in blue, and mutations in red.

The RBD structure of the SARS-CoV-2 S protein complexed with the hACE2 PD was obtained from the Protein Data Bank (PDB) with PDB id 6LZG.^30^ The missing residues of this WT structure were added by the CHARMM-GUI webserver.^31−34^ Four glycans are located at residues 53, 90, and 322 of the hACE2 PD and at residue 343 of the viral RBD. In this work, we adopted two glycan schemes that were created using the CHARMM-GUI web server. In a homogeneous setup, all glycans are of the same type, while in a heterogeneous setup, glycan sites have different glycans^35,36^ (Figure 1). For example, a large oligo-mannose glycan (M8) is chosen in the homogenous scheme because the experiment shows that high-mannose is one of the popular glycan types of SARS-CoV-2 spike protein and human ACE2 protein.^35,37^ Furthermore, the SARS-CoV-2 strain can bind to high mannose glycan in the human body,^38^ and blocking glycan synthesis at the high mannose stage reduces ACE2 binding.^39^ Therefore, we investigated the effect of this type of glycans on the binding of RBD and ACE2.

The Omicron variant was generated from WT RBD using the CHARMM-GUI webserver. In total, we have four complexes with two sets of glycans for each variant. The Zn ion in the hACE2 PD of the original structure was retained. The original structures for the WT and Omicron are shown in Figure 1. All mutations of Omicron are presented in Figure S1 in Supporting Information.

The AMBER19SB and GLYCAM06j force fields were used to describe proteins and glycans.^40,41^ The systems were solvated in a rectangular box filled with four-point OPC water molecules with a minimum distance of 1.3 nm from the solute to the edge of the box.^42^ We used the AMBER19SB force field and OPC water model because according to AMBER force filed developers Tian *et al.*(43) this choice has better predictive power than other options for modeling sequence-specific behaviors and protein mutations. In addition, combining the AMBER14SB force field and the TIP3P water model, Wu *et al.*(24) showed that the WT and Omicron have compatible binding affinities for hACE2, contrary to most experimental data showing that Omicron binds more strongly.^22,23^ Therefore, the TIP3P model was excluded.

To neutralize the system, Na^+^ and Cl^–^ ions were added, maintaining the salt concentration at a physiological level of 0.15 M.

The GROMACS 2021.3 package was used for MD simulation. The solvated systems were minimized using a steep descent algorithm for structure relaxation. The system was then equilibrated in NVT and then in NPT ensembles at 300 K and 1 atm for 500 ps and 5 ns MD runs, respectively. The v-rescale and Parrinello–Rahman algorithms were utilized to keep constant temperature and pressure, respectively.^44,45^ At the equilibration stage, the heavy atoms of the protein–glycan complexes were restrained by a harmonic potential with a spring constant *k* = 1000 kJ/mol/nm.^2^

To estimate the binding free energy by the MM-PBSA method, for each system, five independent MD simulations with a duration of 200 ns were carried out without restraints at 300 K and 1 atm. We used a cutoff of 1.0 nm for non-bonded interactions. The PME method was used to calculate the electrostatic interaction.^46^

### MM-PBSA Method

In the MM-PBSA method, the binding free energy was obtained using the following equation
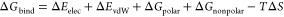
here Δ*E*_elec_ and Δ*E*_vdW_ are the energies of the electrostatic and vdW interactions and Δ*G*_polar_ is the polar solvation energy, which is calculated using Delphi software.^47^ The non-polar solvation energy Δ*G*_nonpolar_ = γΔSASA, where γ = 0.0072 kcal/mol/nm^2^, and SASA is the solvent accessible surface area calculated using the gmx sasa tool in the GROMACS package with a solvent probe radius of 1.4 Å.^48^ The entropy contribution *T*Δ*S* was evaluated according to the method proposed by Duan *et al.*(49)

### Hydrogen Bond

A hydrogen bond is formed if the distance between donor D and acceptor A is less than 0.35 nm, the H–A distance is less than 0.27 nm, and the D–H–A angle is larger than 135°.

### Side chain Contact

A contact between two residues is formed when the distance between the centers of mass of their side chains is ≤6.5 Å.

## Results and Discussion

### Omicron Variant has Higher Binding Affinity than the Wild Type

Root mean square displacement (RMSD) relative to the initial structure was calculated using the atomic coordinates of Cα atoms of the S protein RBD and hACE2 PD. Its time dependence shows that all complexes reached equilibrium after 100 ns (Figures S2 and S3). Therefore, the snapshots collected over the last 100 ns of MD simulation were used for data analysis.

Using the MM-PBSA method, we obtained the binding free energy Δ*G*_bind_ of the WT and Omicron RBD interacting with the hACE2 PD with two glycan models (Table 3). In the case of a homogeneous glycan setup, Omicron has Δ*G*_bind_ (−30.21 ± 5.48 kcal/mol) lower than that of the WT (−18.32 ± 1.62 kcal/mol). This result indicates that the Omicron variant binds to hACE2 more strongly than the WT, which is consistent with the experiment of Cameroni *et al.* and Zhang *et al.*.^22,23^ This conclusion remains also valid for the heterogeneous glycan setup (Table 3).

**Table 3:** Binding Free Energy (kcal/mol) of the WT and Omicron Variant^a^

glycan model	variant	Δ*E*_elec_	Δ*E*_vdW_	Δ*G*_polar_	Δ*G*_nonpolar_	–TΔ*S*	Δ*G*_bind_
homoglycan model	WT	–856.33 ± 3.63	–152.18 ± 9.90	990.29 ± 15.15	–24.27 ± 1.50	24.18 ± 1.29	–18.32 ± 1.62
	Omicron	–1645.73 ± 15.33	–144.86 ± 11.15	1752.76 ± 31.90	–23.58 ± 1.50	31.20 ± 3.06	–30.21 ± 4.48
heteroglycan model	WT	–952.72 ± 17.20	–159.62 ± 6.01	1086.07 ± 14.75	–26.17 ± 0.80	34.87 ± 3.41	–17.57 ± 3.12
	Omicron	–1909.30 ± 18.80	–153.72 ± 6.09	2029.94 ± 40.79	–25.84 ± 2.49	30.94 ± 2.37	–27.97 ± 2.91
no glycans	WT	–778.71 ± 25.20	–93.71 ± 5.12	842.50 ± 27.92	–14.74 ± 0.40	24.78 ± 3.34	–19.88 ± 3.27

a Results were obtained using the MM-PBSA method and snapshots of the last 100 ns from five MD runs. Errors are standard deviations. The last row refers to the WT without glycans.

Experiments showed that *K*_D_ of RBD–PD complexes falls in the nM range (Table 1), which corresponds to Δ*G*_bind_ ∼ −12 kcal/mol. Therefore, the absolute value of the binding free energy predicted by the MM-PBSA method is much larger than the experimental value, implying that this method is good for evaluating the relative binding free energies but not their absolute value. The same has been mentioned in previous studies.^50,51^ By combining coarse-grained models^52^ with umbrella sampling, reasonable results can be obtained for the absolute value of Δ*G*_bind_ and *K*_D_,^53^ but coarse-grained modeling is not sensitive enough to describe effects of mutations.

Using the same MM-PBSA method and AMBER19SB force field, Wu *el al*(18) showed that the WT and Omicron have comparable binding affinities, contradicting our results. Here are some of the reasons for this difference: we used the four-point OPC water model, while Wu *et al.* used TIP3P; we took into account glycans that were neglected by Wu *et al.*

### Electrostatic Interaction Plays a Crucial Role in the Stability of the RBD-PD Complex

The electrostatic interaction between S protein and ACE2 dominates over the vdW interaction in all complexes of hACE2 and SARS-CoV-2 RBD (Table 3). For the WT and homogeneous glycan model, *E*_elec_ = −856 kcal/mol, which is much less than *E*_vdW_ = −152 kcal/mol. For the Omicron variant, the role of the electrostatic interaction becomes even more pronounced because for the same glycan model, we have *E*_elec_ = −1645 kcal/mol and *E*_vdW_ = −145 kcal/mol (Table 3). This conclusion is also valid for the heterogeneous glycan model. The dominant role of the electrostatic interaction is related to the fact that both PD of hACE2 and RBD are charged. The charge of hACE2 PD is −27e, while the charge of WT RBD is +3e and of Omicron RBD is +6e. Thus, the higher binding affinity of Omicron is due to the increased attractive electrostatic interaction.

### Impact of the Glycan Model on the Interaction Energy Between RBD and hACE2 PD

We divided the interaction energies between the viral RBD and hACE2 PD into protein and glycan parts (Table S1). In both glycan models, the energy contribution of proteins dominates over that of glycans.

**Figure 2:**
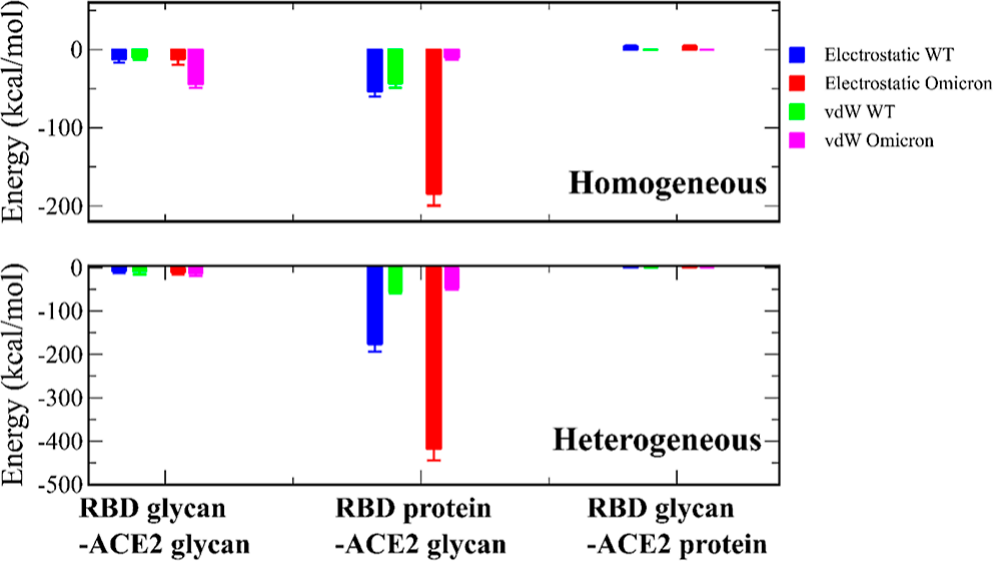
Energy of RBD glycan-hACE2 glycan, RBD protein-hACE2 glycan, and RBD glycan-hACE2 protein interactions.

In the homogeneous glycan model, hACE2 glycans have a stronger vdW interaction with WT RBD (−42.88 kcal/mol) than Omicron (−10.34 kcal/mol) (Table S1, Figure 2). The electrostatic interaction between hACE2 glycans and Omicron RBD glycans is repulsive (41.59 kcal/mol), while hACE2 glycans have an attractive electrostatic interaction with the WT RBD glycans (−12.36 kcal/mol). The interaction energy of RBD glycans with hACE2 protein (≈ 50 kcal/mol) is higher than that of WT glycans (≈ 4 kcal/mol), which implies that RBD glycans reduces the binding affinity of Omicron to a greater extent than the WT.

In the case of the heterogeneous glycan model, the interaction energies between RBD and hACE2 glycans are equivalent within errors for the WT and Omicron (Table S1, Figure 2). Similarly, the difference in interactions between RBD glycans and hACE2 protein is insignificant for the WT and Omicron. The interaction energy between RBD and hACE2 glycans is larger than that in the homogeneous model. The protein part of the Omicron RBD has a significantly lower non-bonded interaction energy with hACE2 glycans (−465 kcal/mol) than the WT (−230 kcal/mol) (Figure 2). These results suggest that the influence of glycan models on specific components of the interaction energy between the RBD and hACE2 is important. However, the effect of glycan models on the difference in the binding affinity of SARS-CoV-2 variants to hACE2 is insignificant (Table 3).

We calculated the percentage of SASA of glycans in relation to the total SASA (Table S2). The area covered by hACE2 glycans in the Omicron case is clearly larger than that in WT in both glycosylation models. The enhanced SASA ratio indicates greater exposure of ACE2 glycans to solvent in the case of Omicron. However, RBD glycans have the same coverage in both variants, which is probably due to the fact that the RBD has only one glycosylation site at residue N343. Thus, the Omicron variant strongly alters the orientation of glycan molecules of hACE2 but not the RBD.

### Important Residues in Binding of Viral RBD and hACE2 PD: Strong Effects of Glycan Models and Importance of Electrostatic Interaction

**Figure 3:**
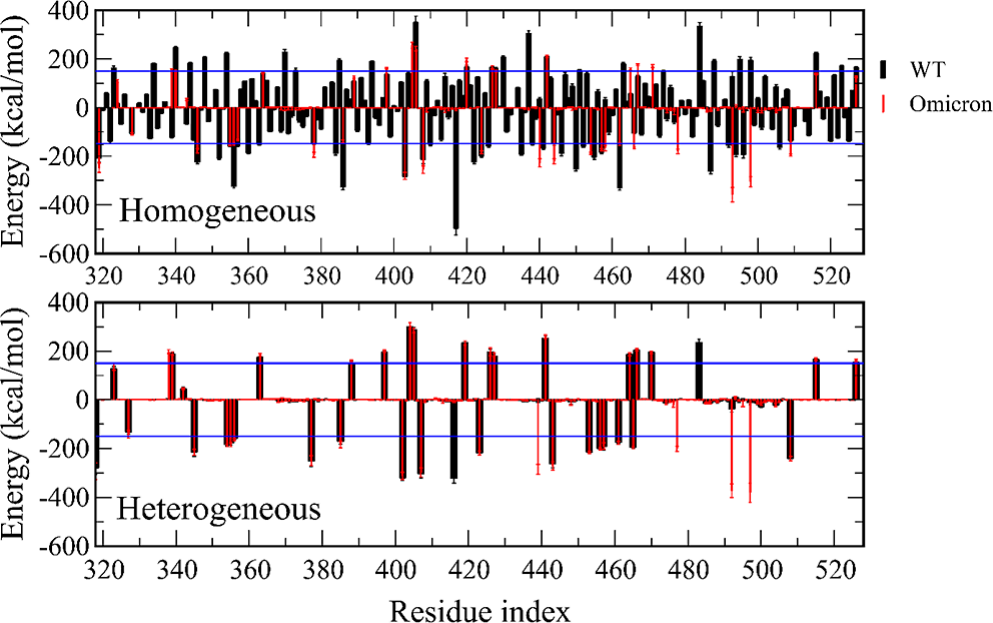
Interaction energy for individual residues. The blue line refers to 150 and -150 kcal/mol.

We calculated the contribution of RBD residues to the interaction energy with hACE2 for the WT and Omicron (Figure 3). Residues that have an absolute value of the interaction energy ≥150 kcal/mol are listed in Table 4 and their positions are shown in Figure S4. Glycan models have a noticeable effect on the per-residue distribution of interaction energies (Figure 3). In the homogeneous glycan model, the number of RBD residues that have an interaction energy below −150 kcal/mol is 26 for the WT and 18 for Omicron, while in the heterogeneous model, these numbers are 18 and 21, respectively (Table 4). The number of residues with an interaction energy exceeding 150 kcal/mol is also different for the two glycan models.

**Table 4:** Residues of RBD That Have an Absolute Interaction Energy with hACE2 PD ≥ 150 kcal/mol

glycan model	system	residues that have an interaction energy ≤ −150 kcal/mol	residues that have an interaction energy ≥150kcal/mol
homogeneous	WT	R319, R346, R355, K356, R357, N360, P384, K386, R403, R408, K417, N422, K424, P426, L441, G446, N450, L452, L455, R457, K462, N487, L492, S494, G496, Q506	T323, N334, E340, N354, N370, S373, T385, N394, E406, D420, D427, D428, T430, N437, D442, Y451, P463, E484, C488, Y495, Q498, E516, T523, P527
	Omicron	R319, R346, R355, K378, R403, R408, K424, K440, K444, R454, R457, K458, K462, R466, K478, R493, R498, R509	D339, E340, D398, D405, E406, D420, D427, D428, D442, E465, D467, E471
heterogeneous	WT	R319, R346, R355, K356, R357, K378, K386, R403, R408, K417, K424, K444, R454, R457, K458, K462, R466, R509	E340, D364, D389, D398, D405, E406, D420, D427, D428, D442, E465, D467, E471, E484, E516, P527
	Omicron	R319, R346, R355, K356, R357, K378, K386, R403, R408, K424, K440, K444, R454, R457, K458, K462, R466, K478, R493, R498, R509	D339, E340, D364, D389, D398, D405, E406, D420, D427, D428, D442, D465, E467, D471, E516, P527

**Figure 4:**
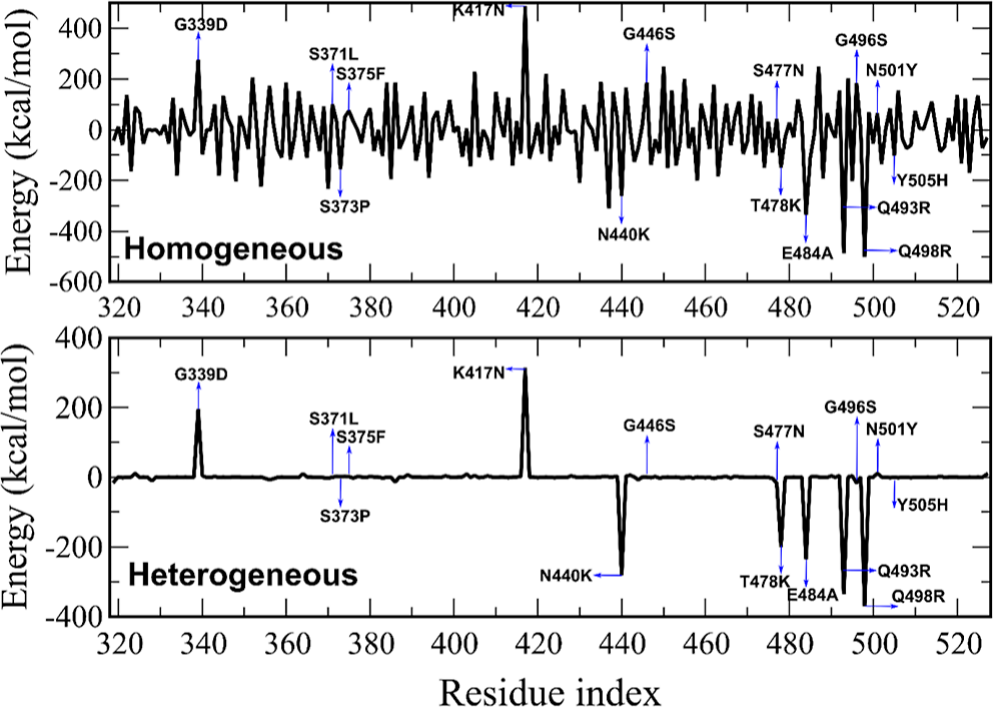
Difference between the interaction energies of Omicron and WT residues (*E*_Omicron_ – *E*_WT_) for homogeneous (upper) and heterogeneous (bottom) glycan models.

To investigate the effect of mutations in Omicron, the difference between the per-residue energies of Omicron and WT (*E*_Omicron_ – *E*_WT_) was calculated (Figure 4). In the homogeneous glycan model, the difference between the WT and Omicron is seen for many RBD residues with large energy fluctuations. However, in the heterogeneous model, the energy difference has a sharp peak at a much lower number of mutations, again showing that the glycan model drastically affects the interaction energies of RBD with hACE2 across residues.

For the homogeneous glycan model, not only mutated residues but also other RBD residues contribute to the different stability of the WT and Omicron (Figure 4). Mutations G339D, K417N, G446S, and G496S destabilize the complex, while S373P, N440K, T487K, E484A, Q493R, Q498R, and Y505H stabilize it, highlighting the importance of mutations that change charge. Namely, G339D and K417N reduce the net charge, resulting in a weaker interaction between Omicron and hACE2. In contrast, N440K, T487K, E484A, Q493R, Q498R, and Y505H increase the total charge, promoting an attractive interaction of the RBD with the negatively charged hACE2.

In the heterogeneous glycan model, the pattern of per-residue interaction energies is much simpler than that in the homogeneous case (Figure 4). Interestingly, only the mutated residues in the RBD play a primary role in the energy difference between the WT and Omicron as in the homogeneous setup, the mutations G339D and K417N weaken the interaction between RBD and hACE2 by increasing the non-bonded interaction energy in Omicron compared to WT. In addition, mutations N440K, T478K, E484A, Q493R, Q498R enhance the binding affinity, but the mutations S371L, S373P, S375F, G446S, S477N, G496S, N501Y, and Y505H have a negligible effect. The effect of G339D and K417N mutations on the binding energy is less than that caused by N440K, T478K, E484A, Q493R, and Q498R mutations, leading to the stronger binding of Omicron to hACE2.

Finally, we try to understand why WT and Omicron are so different in the homogeneous model but look almost identical in the heterogeneous model (Figures 3 and 4). The difference between the two glycan models can be explained taking into account the fact that the stability of the RBD-ACE2 complex is governed by electrostatic interaction. Since in the presence of glycans, an additional electrostatic interaction between RBD and ACE2-glycans dominates (Figure 2), we will focus on this interaction. In the heterogeneous model, this interaction is strong and attractive because the charge of ACE2-glycans is large and negative (−2.4 e) (see Table S3). Therefore, this strong interaction should not significantly change the pattern of per-residue interaction energies of RBD-ACE2 (Figures 3 and 4) because for RBD-ACE2, the electrostatic interaction is also dominant without glycans.

In contrast, for the homogeneous model, the electrostatic interaction between RBD and ACE2-glycans is weak because the charge of ACE2-glycans is small (0.6 e) (Table S3). Therefore, the contribution of this term to the per-residue interaction energies of RBD-ACE2 can be considered as small fluctuations that lead to the noisy patterns shown in Figures 3 and 4.

### Important Residues at the RBD-hACE2 Interface

To investigate the contribution of residues located at the RBD-hACE2 interface to the complex stability, we calculated the interaction energy of RBD residues that form side-chain contact with hACE2. In the homogeneous glycan model, the number of these residues are 20 and 18 for the WT and Omicron, respectively (Figure S5). For the heterogeneous setup, we have 25 and 19 residues for the WT and Omicron, respectively. Using snapshots from the last 100 ns of MD simulation, we can show that the population of side-chain contacts formed by these residues with hACE2 varies from a few % to ≈50% (Figure S5). This population is sensitive to glycan models, but is generally greater for Omicron than for WT, implying a higher binding affinity of Omicron.

**Figure 5:**
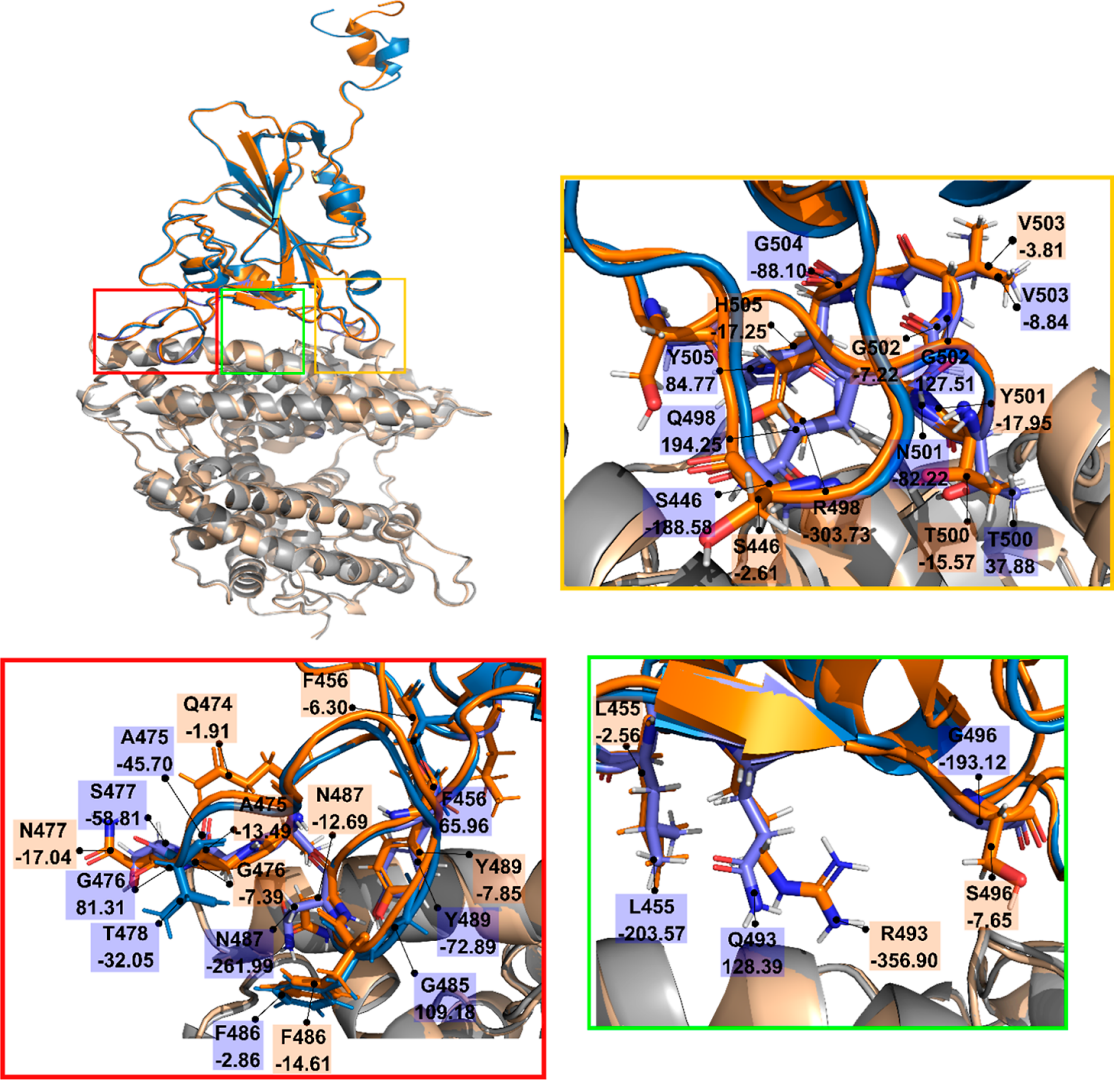
Alignment of the WT RBD (blue)-hACE2 (gray) and Omicron RBD (orange)-hACE2 (wheat) complexes. For clarity glycans have been removed. RBD residues that have a side-chain contact with hACE2 residues are enclosed in large red, green, and yellow boxes for the homogeneous glycan model. The labels of RBD residues and their interaction energy with hACE2 are shown in small blue and orange rectangles for the WT and Omicron, respectively.

**Figure 6:**
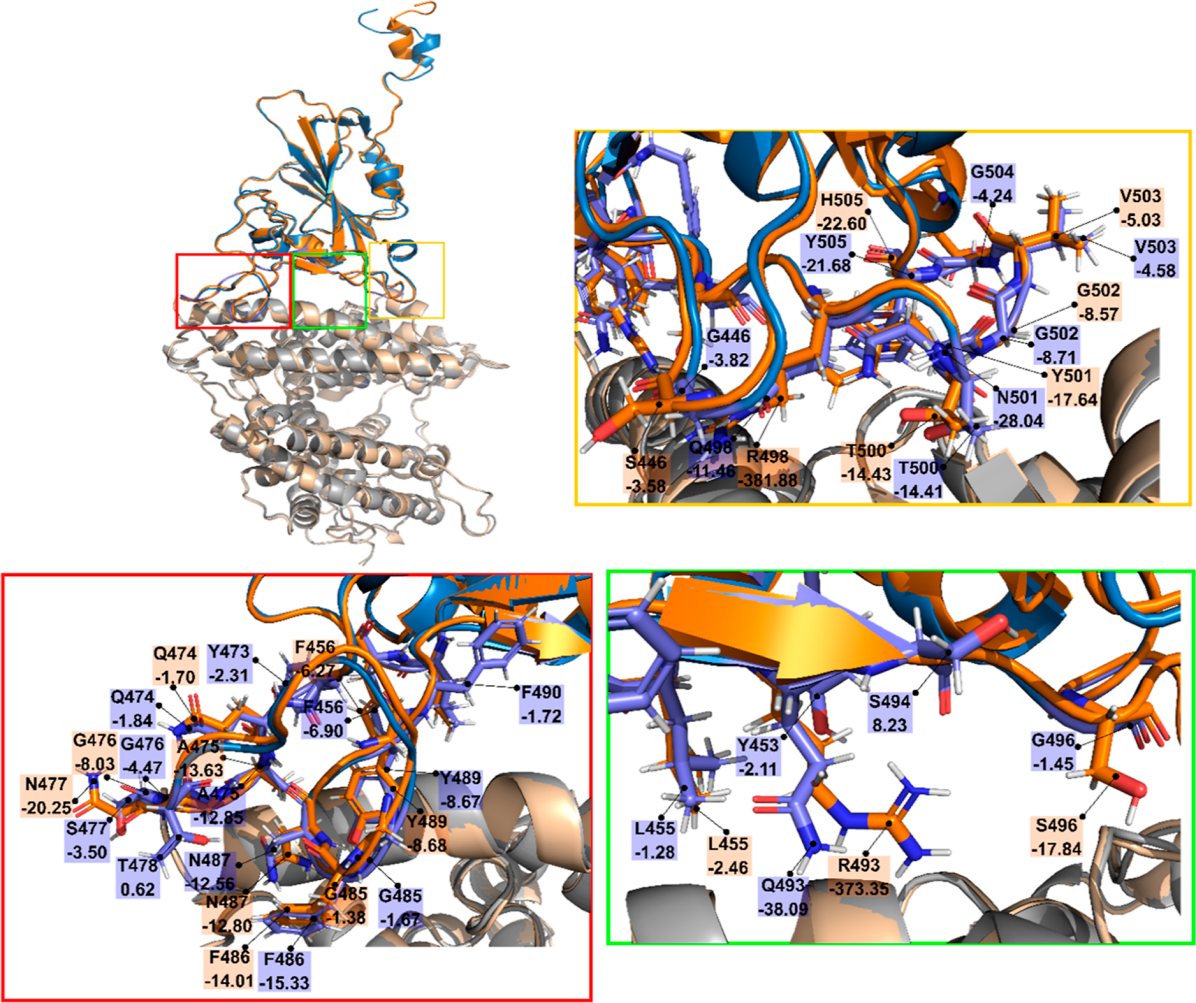
Same as in Figure 5 but for the heterogeneous glycan model.

For clarity, we divide these residues into red, green, and yellow regions as shown in Figures 5 and 6 (see also Figure S5). With the homogeneous glycan model, the contribution of these regions to the total energy is −212.38 and −508.15 kcal/mol for the WT and Omicron, respectively. In the heterogeneous glycan scheme, these contributions are −202.84 and −870.43 kcal/mol. Therefore, the contribution of the interface area to the stability of RBD–hACE2 is significant.

For the homogenous glycan model, the red region of Omicron (Figure 5) has a weaker interaction with hACE2 (−71.20 kcal/mol) compared to WT (−217.85 kcal/mol) (Table 5). This effect corresponds to a decrease in the hydropathy index from 3.1 for the WT to −2.1 for Omicron (Table 5), indicating that the red region of the WT is more hydrophobic than Omicron. In particular, the S477N mutation attenuates the interaction between the RBD and hACE2 from −58.81 (WT) to −17.04 kcal/mol (Omicron) (Figure 5). In the green region, the mutation Q493R dramatically increases the interaction between the two molecules from 128.39 (WT) to −356.90 kcal/mol (Omicron). This effect occurs because the R amino acid has a positive charge (+e) while the Q amino acid is neutral. In contrast, the G496S mutation weakens the interaction (Figure 5). L455 is not mutated, but in WT, this residue has a lower interaction energy than in Omicron. Thus, the total interaction energy of residues in the green region of WT (Figure 5) is −268.30 kcal/mol which is higher than −367.11 kcal/mol of Omicron. In the yellow region (Figure 5), the mutations Q498R and Y505H enhance the interaction between RBD and hACE2 while N501Y weakens it. Like the Q493R mutation, the great energy change by Q498R is due to the positive charge of the R amino acid. The energy of the residues in the yellow region is 76.67 kcal/mol in the WT, while for the Omicron variant, it is −368.14 kcal/mol. Therefore, although Omicron has a weaker interaction in the red region, it interacts much strongly with hACE2 in other regions, which explains why *K*_D_ of Omicron is lower compared to that of WT, as observed in experiments of Cameroni *et al.* and Zhang *et al.*(22,23)

**Table 5:** Average Total Interaction Energy, Total Hydropathy,^60^ and Total Charge of RBD residues That Have a Side-chain Contact with hACE2^a^

glycan model	variant	region	average total energy (kcal/mol)	hydropathy	charge (e)
homogeneous	WT	red	–217.85	3.1	0
		green	–268.30	–0.1	0
		yellow	76.67	–6.4	0
	Omicron	red	–81.28	–2.1	0
		green	–367.11	–1.5	1
		yellow	–368.14	–6.7	1
heterogeneous	WT	red	–71.20	–1.7	0
		green	–34.70	–2.2	0
		yellow	–96.94	–9.1	0
	Omicron	red	–58.73	–5.2	0
		green	–357.97	–1.5	1
		yellow	–453.73	–6.7	1

a Data are divided into 3 regions red, green, and yellow.

For heterogeneous glycans, mutations in Omicron have the same effect in the red, green, and yellow regions as in the homogeneous setting (Figure 6). The contribution to the complex stability from the red region of Omicron is lesser than that from the WT, while the opposite effect takes place in the green and yellow regions (Table 5). Similar to the homogeneous glycan model, the S477N mutation promotes the RBD–hACE2 association by reducing the interaction energy from −3.50 to −20.25 kcal/mol. Furthermore, the total interaction energy of the red region in Omicron (−58.73 kcal/mol) is higher than that of the WT (−71.2 kcal/mol). In the green region, the Q493R mutation enhances the stability of the complex since the interaction energy of R493 is −373.35 kcal/mol for Omicron *versus* −38.09 kcal/mol of Q493 for WT. Therefore, due to this mutation in the green region, Omicron has a notably lower interaction energy than WT (Table 5). In the yellow region, Q498R plays the same role as in the green area.

Thus, the interaction energies of residues at the RBD-hACE2 interface depend on glycan models, but both studied models exhibit the same trend, which is that compared to the WT, Omicron has a higher interaction energy in the red region, while a lower energy is observed in the green and yellow regions. This suggests that due to evolution, a new SARS-CoV-2 variant may have a higher binding affinity, resulting in faster infection than Omicron should it has mutations in the red region.

### Glycans Have a Little Effect on the RBD-hACE2 Binding Free Energy

So far, we have discussed four systems with glycans covering both the viral RBD and hACE2 PD. In order to access the influence of glycans on the binding free energy, we performed five independent MD runs of 200 ns each for the RBD-hACE2 WT without glycans. Using the last 100 ns snapshots of the five MD trajectories and the MM-PBSA method, we obtained Δ*G*_bind_ shown in the last row of Table 3. For the WT without glycans, we have Δ*G*_bind_ = −19.88 ± 3.27 kcal/mol, which does not differ greatly from −18.32 ± 1.62 to −17.57 ± 3.12 kcal/mol of the WT surrounded by homogeneous and heterogeneous glycans, respectively. Thus, glycans have weak influence on the stability of RBD-hACE2 WT. This conclusion is expected to be valid for the Omicron variant.

It must be emphasized that the effect of glycan on viral attachment to host cells is not entirely clear. Our MM-PBSA result is consistent with the experimental study of Allen *et al.* showing that the glycans play a limited role in the SARS-CoV-2 RBD and human ACE2 binding.^54^ However, computational studies of Mehdipour and Hummer,^55^ Zhao *et al.*,^37^ and Barros *et al.*(56) demonstrated that glycans in ACE2 are important in RBD binding. The difference between our results and those of these groups may be due to different force fields and systems used. Although all the groups used the same force field for glycans, for protein and ions, we used Amber19SB and the OPC water model, Mehdipour *et al.*(55)—CHARMM36m and TIP3P, and Zhao *et al.*(37)—Amber14SB and TIP3P, while Barros *et al.*(56)—CHARMM36 and TIP3P. Our group and Barros *et al.*(56) studied the same RBD–ACE2 complex without membranes. However, Mehdipour *et al.*(55) considered B0AT1–ACE2–RBD in complex with the viral membrane, while Zhao *et al.*(37) considered that the RBD dimer interacts with homodimeric membrane-anchored ACE2. Therefore, more experimental and computational work is needed to elucidate the role of glycans in RBD-ACE2 stability.

## Conclusions

Using MD simulation and the MM-PBSA method, we obtained the binding free energy of the WT and Omicron variant of SARS-CoV-2 to the hACE2 PD, which shows that Omicron binds more tightly than the WT. This result can be invoked to explain the high infection rate of the Omicron variant. The electrostatic interaction was found to rule the stability of the viral RBD–hACE2 PD complex. Since hACE2 PD is negatively charged, the increase in binding affinity is due in part to an increase in the overall charge of the RBD from +3e (WT) to +6e (Omicron).

The influence of the glycan models studied in this work is twofold: the per-residue interaction energies are sensitive to the glycan scheme, but the binding free energy does not depend on it. Whether this conclusion holds for other glycan models or not requires a further study.

We demonstrated that N440K, T478K, E484A, Q493R, and Q498R mutations play a crucial role in the high binding affinity of Omicron to human cells. After a detailed analysis of residues located at the human cell–virus interface, we predict that the emergence of a new variant with a higher infection rate compared to that of Omicron is still possible. Such a variant should have mutations in the red interface region indicated in Figures 5 and 6.

After submitting the article, we learned that several cryoEM structures of the Omicron spike protein have been published including structures with PDB ID 7TEI,^57^ 7TLY,^58^ and 7T9J.^59^ We calculated RMSD between our Omicron RBD model generated with CHARMM-GUI and these structures (Figure S6). We got RMSD = 15.11, 4.55, and 8.09 Å for 7TEI, 7TLY, and 7T9J respectively. The main difference in RMSD is related to the N-terminus (Figure S6), which does not belong to the RBD–ACE2 interface (Figure 5). Thus, we expect that the use of the cryoEM structure should not change our results, at least qualitatively.

## Supporting Information Available

Contributions of non-bonded interaction energy (kcal/mol) between protein–glycan and protein–protein of the hACE2–RBD complexes; ratio (%) of SASA of glycan molecules binding to the hACE2 and RBD in relation to total SASA; charge (measured in e) on ACE2, RBD, ACE2-glycans, and RBD-glycans; mutations in the RBD of Omicron; time dependence of Cα RMSD of the WT RBD–hACE2 complex with the homogeneous (upper) and heterogeneous (bottom) models; time dependence of Cα RMSD of the Omicron RBD–hACE2 complex with homogeneous (upper) and heterogeneous (bottom) glycan models; residues of RBD that have an absolute interaction energy with hACE2 ≥ 150 kcal/mol; and population of side-chain contacts formed by RBD residues with hACE2 at the interface (PDF)

## References

[ref1] Gorbalenya A. E.; Baker S. C.; Baric R. S.; de Groot R. J.; Drosten C.; Gulyaeva A. A.; Haagmans B. L.; Lauber C.; Leontovich A. M.; Neuman B. W.; Penzar D.; Perlman S.; Poon L. L. M.; Samborskiy D. V.; Sidorov I. A.; Sola I.; Ziebuhr J. Coronaviridae Study Group of the International Committee on Taxonomy of, V. The species Severe acute respiratory syndrome-related coronavirus: classifying 2019-nCoV and naming it SARS-CoV-2. Nat. Microbiol. 2020, 5, 536–544. 32123347 10.1038/s41564-020-0695-z PMC7095448

[ref2] Dong E.; Du H.; Gardner L. An interactive web-based dashboard to track COVID-19 in real time. Lancet Infect. Dis. 2020, 20, 533–534. 10.1016/s1473-3099(20)30120-1. 32087114 PMC7159018

[ref3] Yang J.; Petitjean S. J.; Koehler M.; Zhang Q.; Dumitru A. C.; Chen W.; Derclaye S.; Vincent S. P.; Soumillion P.; Alsteens D. Molecular interaction and inhibition of SARS-CoV-2 binding to the ACE2 receptor. Nat. Commun. 2020, 11, 4541 10.1038/s41467-020-18319-6. 32917884 PMC7486399

[ref4] Iyer A. S.; Jones F. K.; Nodoushani A.; Kelly M.; Becker M.; Slater D.; Mills R.; Teng E.; Kamruzzaman M.; Garcia-Beltran W. F. Persistence and decay of human antibody responses to the receptor binding domain of SARS-CoV-2 spike protein in COVID-19 patients. Sci. Immunol. 2020, 5, abe0367 10.1126/sciimmunol.abe0367. PMC7857394 33033172

[ref5] Wang P.; Nair M. S.; Liu L.; Iketani S.; Luo Y.; Guo Y.; Wang M.; Yu J.; Zhang B.; Kwong P. D.; Graham B. S.; Mascola J. R.; Chang J. Y.; Yin M. T.; Sobieszczyk M.; Kyratsous C. A.; Shapiro L.; Sheng Z.; Huang Y.; Ho D. D. Antibody resistance of SARS-CoV-2 variants B.1.351 and B.1.1.7. Nature 2021, 593, 130–135. 10.1038/s41586-021-03398-2. 33684923

[ref6] Premkumar L.; Segovia-Chumbez B.; Jadi R.; Martinez D. R.; Raut R.; Markmann A.; Cornaby C.; Bartelt L.; Weiss S.; Park Y.; Edwards C. E.; Weimer E.; Scherer E. M.; Rouphael N.; Edupuganti S.; Weiskopf D.; Tse L. V.; Hou Y. J.; Margolis D.; Sette A.; Collins M. H.; Schmitz J.; Baric R. S.; de Silva A. M. The receptor binding domain of the viral spike protein is an immunodominant and highly specific target of antibodies in SARS-CoV-2 patients. Sci. Immunol. 2020, 5, eabc8413 10.1126/sciimmunol.abc8413. 32527802 PMC7292505

[ref7] Jackson C. B.; Farzan M.; Chen B.; Choe H. Mechanisms of SARS-CoV-2 entry into cells. Nat. Rev. Mol. Cell Biol. 2022, 23, 3–20. 10.1038/s41580-021-00418-x. 34611326 PMC8491763

[ref8] Lan J.; Ge J.; Yu J.; Shan S.; Zhou H.; Fan S.; Zhang Q.; Shi X.; Wang Q.; Zhang L.; Wang X. Structure of the SARS-CoV-2 spike receptor-binding domain bound to the ACE2 receptor. Nature 2020, 581, 215–220. 10.1038/s41586-020-2180-5. 32225176

[ref9] Shang J.; Ye G.; Shi K.; Wan Y.; Luo C.; Aihara H.; Geng Q.; Auerbach A.; Li F. Structural basis of receptor recognition by SARS-CoV-2. Nature 2020, 581, 221–224. 10.1038/s41586-020-2179-y. 32225175 PMC7328981

[ref10] Wrapp D.; Wang N.; Corbett K. S.; Goldsmith J. A.; Hsieh C.-L.; Abiona O.; Graham B. S.; McLellan J. S. Cryo-EM structure of the 2019-nCoV spike in the prefusion conformation. Science 2020, 367, 1260–1263. 10.1126/science.abb2507. 32075877 PMC7164637

[ref11] Walls A. C.; Park Y.-J.; Tortorici M. A.; Wall A.; McGuire A. T.; Veesler D. Structure, Function, and Antigenicity of the SARS-CoV-2 Spike Glycoprotein. Cell 2020, 181, 281–292. 10.1016/j.cell.2020.02.058. 32155444 PMC7102599

[ref12] Nguyen H. L.; Lan P. D.; Thai N. Q.; Nissley D. A.; O’Brien E. P.; Li M. S. Does SARS-CoV-2 Bind to Human ACE2 More Strongly Than Does SARS-CoV?. J. Phys. Chem. B 2020, 124, 7336–7347. 10.1021/acs.jpcb.0c04511. 32790406 PMC7433338

[ref13] Kim S.; Liu Y.; Lei Z.; Dicker J.; Cao Y.; Zhang X. F.; Im W. Differential Interactions between Human ACE2 and Spike RBD of SARS-CoV-2 Variants of Concern. J. Chem. Theory Comput. 2021, 17, 7972–7979. 10.1021/acs.jctc.1c00965. 34856802 PMC8672429

[ref14] Cao W.; Dong C.; Kim S.; Hou D.; Tai W.; Du L.; Im W.; Zhang X. F. Biomechanical characterization of SARS-CoV-2 spike RBD and human ACE2 protein-protein interaction. Biophys. J. 2021, 120, 1011–1019. 10.1016/j.bpj.2021.02.007. 33607086 PMC7886630

[ref15] Gao K.; Wang R.; Chen J.; Cheng L.; Frishcosy J.; Huzumi Y.; Qiu Y.; Schluckbier T.; Wei X.; Wei G. W. Methodology-Centered Review of Molecular Modeling, Simulation, and Prediction of SARS-CoV-2. Chem. Rev. 2022, 10.1021/acs.chemrev.1c00965. PMC9159519 35594413

[ref16] World Health Organization. Classification of Omicron (B. 1.1. 529): SARS-CoV-2 variant of concern; WHO, 2021.

[ref17] Gao S.-J.; Guo H.; Luo G. Omicron variant (B.1.1.529) of SARS-CoV-2, a global urgent public health alert!. J. Med. Virol. 2022, 94, 1255 10.1002/jmv.27491. 34850421 PMC9015397

[ref18] Tareq A. M.; Emran T. B.; Dhama K.; Dhawan M.; Tallei T. E. Impact of SARS-CoV-2 delta variant (B.1.617.2) in surging second wave of COVID-19 and efficacy of vaccines in tackling the ongoing pandemic; Human Vaccines & Immunotherapeutics, 2021; pp 1–2. 10.1080/21645515.2021.1963601 PMC8425453 34473593

[ref19] Grant R.; Charmet T.; Schaeffer L.; Galmiche S.; Madec Y.; Von Platen C.; Chény O.; Omar F.; David C.; Rogoff A.; Paireau J.; Cauchemez S.; Carrat F.; Septfons A.; Levy-Bruhl D.; Mailles A.; Fontanet A. Impact of SARS-CoV-2 Delta variant on incubation, transmission settings and vaccine effectiveness: Results from a nationwide case-control study in France. Lancet 2022, 13, 100278. 10.1016/j.lanepe.2021.100278 PMC8616730 34849500

[ref20] Pouwels K. B.; Pritchard E.; Matthews P. C.; Stoesser N.; Eyre D. W.; Vihta K.-D.; House T.; Hay J.; Bell J. I.; Newton J. N.; Farrar J.; Crook D.; Cook D.; Rourke E.; Studley R.; Peto T. E. A.; Diamond I.; Walker A. S. Effect of Delta variant on viral burden and vaccine effectiveness against new SARS-CoV-2 infections in the UK. Nat. Med. 2021, 27, 2127–2135. 10.1038/s41591-021-01548-7. 34650248 PMC8674129

[ref21] Karim S. S. A.; Karim Q. A. Omicron SARS-CoV-2 variant: a new chapter in the COVID-19 pandemic. Lancet 2021, 398, 2126–2128. 10.1016/s0140-6736(21)02758-6. 34871545 PMC8640673

[ref22] Cameroni E.; Bowen J. E.; Rosen L. E.; Saliba C.; Zepeda S. K.; Culap K.; Pinto D.; VanBlargan L. A.; De Marco A.; di Iulio J.; Zatta F.; Kaiser H.; Noack J.; Farhat N.; Czudnochowski N.; Havenar-Daughton C.; Sprouse K. R.; Dillen J. R.; Powell A. E.; Chen A.; Maher C.; Yin L.; Sun D.; Soriaga L.; Bassi J.; Silacci-Fregni C.; Gustafsson C.; Franko N. M.; Logue J.; Iqbal N. T.; Mazzitelli I.; Geffner J.; Grifantini R.; Chu H.; Gori A.; Riva A.; Giannini O.; Ceschi A.; Ferrari P.; Cippà P. E.; Franzetti-Pellanda A.; Garzoni C.; Halfmann P. J.; Kawaoka Y.; Hebner C.; Purcell L. A.; Piccoli L.; Pizzuto M. S.; Walls A. C.; Diamond M. S.; Telenti A.; Virgin H. W.; Lanzavecchia A.; Snell G.; Veesler D.; Corti D. Broadly neutralizing antibodies overcome SARS-CoV-2 Omicron antigenic shift. Nature 2020, 602, 664 10.1038/s41586-021-04386-2. PMC9531318 35016195

[ref23] Zhang X.; Wu S.; Wu B.; Yang Q.; Chen A.; Li Y.; Zhang Y.; Pan T.; Zhang H.; He X. SARS-CoV-2 Omicron strain exhibits potent capabilities for immune evasion and viral entrance. Signal Transduction Targeted Ther. 2021, 6, 430 10.1038/s41392-021-00852-5. PMC8678971 34921135

[ref24] Wu L.; Zhou L.; Mo M.; Liu T.; Wu C.; Gong C.; Lu K.; Gong L.; Zhu W.; Xu Z. SARS-CoV-2 Omicron RBD shows weaker binding affinity than the currently dominant Delta variant to human ACE2. Signal Transduction Targeted Ther. 2022, 7, 8 10.1038/s41392-021-00863-2. PMC8727475 34987150

[ref25] Chan K. K.; Dorosky D.; Sharma P.; Abbasi S. A.; Dye J. M.; Kranz D. M.; Herbert A. S.; Procko E. Engineering human ACE2 to optimize binding to the spike protein of SARS coronavirus 2. Science 2020, 369, 1261–1265. 10.1126/science.abc0870. 32753553 PMC7574912

[ref26] Chen J.; Wang R.; Gilby N. B.; Wei G.-W. Omicron Variant (B.1.1.529): Infectivity, Vaccine Breakthrough, and Antibody Resistance. J. Chem. Inf. Model. 2022, 62, 412–422. 10.1021/acs.jcim.1c01451. 34989238 PMC8751645

[ref27] Kumar S.; Thambiraja T. S.; Karuppanan K.; Subramaniam G. Omicron and Delta variant of SARS-CoV-2: A comparative computational study of spike protein. J. Med. Virol. 2022, 94, 1641 10.1002/jmv.27526. 34914115

[ref28] Omotuyi O.; Olubiyi O.; Nash O.; Afolabi E.; Oyinloye B.; Fatumo S.; Femi-Oyewo M.; Bogoro S. SARS-CoV-2 Omicron spike glycoprotein receptor binding domain exhibits super-binder ability with ACE2 but not convalescent monoclonal antibody. Comput. Biol. Med. 2022, 142, 105226 10.1016/j.compbiomed.2022.105226. 35066447 PMC8739363

[ref29] Kim S.; Liu Y.; Ziarnik M.; Cao Y.; Zhang X. F.; Im W. Binding of Human ACE2 and RBD of Omicron Enhanced by Unique Interaction Patterns Among SARS-CoV-2 Variants of Concern; bioRxiv, 2022. 10.1002/jcc.27025 PMC9825653 36398990

[ref30] Wang Q.; Zhang Y.; Wu L.; Niu S.; Song C.; Zhang Z.; Lu G.; Qiao C.; Hu Y.; Yuen K.-Y.; Wang Q.; Zhou H.; Yan J.; Qi J. Structural and Functional Basis of SARS-CoV-2 Entry by Using Human ACE2. Cell 2020, 181, 894–904. 10.1016/j.cell.2020.03.045. 32275855 PMC7144619

[ref31] Jo S.; Kim T.; Iyer V. G.; Im W. CHARMM-GUI: A web-based graphical user interface for CHARMM. J. Comput. Chem. 2008, 29, 1859–1865. 10.1002/jcc.20945. 18351591

[ref32] Jo S.; Song K. C.; Desaire H.; MacKerell A. D. Jr.; Im W. Glycan reader: Automated sugar identification and simulation preparation for carbohydrates and glycoproteins. J. Comput. Chem. 2011, 32, 3135–3141. 10.1002/jcc.21886. 21815173 PMC3188666

[ref33] Park S.-J.; Lee J.; Patel D. S.; Ma H.; Lee H. S.; Jo S.; Im W. Glycan Reader is improved to recognize most sugar types and chemical modifications in the Protein Data Bank. Bioinformatics 2017, 33, 3051–3057. 10.1093/bioinformatics/btx358. 28582506 PMC5870669

[ref34] Park S.-J.; Lee J.; Qi Y.; Kern N. R.; Lee H. S.; Jo S.; Joung I.; Joo K.; Lee J.; Im W. CHARMM-GUIGlycan Modelerfor modeling and simulation of carbohydrates and glycoconjugates. Glycobiology 2019, 29, 320–331. 10.1093/glycob/cwz003. 30689864 PMC6422236

[ref35] Shajahan A.; Archer-Hartmann S.; Supekar N. T.; Gleinich A. S.; Heiss C.; Azadi P. Comprehensive characterization of N- and O- glycosylation of SARS-CoV-2 human receptor angiotensin converting enzyme 2. Glycobiology 2020, 31, 410–424. 10.1093/glycob/cwaa101. PMC7665489 33135055

[ref36] Watanabe Y.; Allen J. D.; Wrapp D.; McLellan J. S.; Crispin M. Site-specific glycan analysis of the SARS-CoV-2 spike. Science 2020, 369, 330–333. 10.1126/science.abb9983. 32366695 PMC7199903

[ref37] Zhao P.; Praissman J. L.; Grant O. C.; Cai Y.; Xiao T.; Rosenbalm K. E.; Aoki K.; Kellman B. P.; Bridger R.; Barouch D. H.; Brindley M. A.; Lewis N. E.; Tiemeyer M.; Chen B.; Woods R. J.; Wells L. Virus-Receptor Interactions of Glycosylated SARS-CoV-2 Spike and Human ACE2 Receptor. Cell Host Microbe 2020, 28, 586–601. 10.1016/j.chom.2020.08.004. 32841605 PMC7443692

[ref38] Byrd-Leotis L.; Lasanajak Y.; Bowen T.; Baker K.; Song X.; Suthar M. S.; Cummings R. D.; Steinhauer D. A. SARS-CoV-2 and other coronaviruses bind to phosphorylated glycans from the human lung. Virology 2021, 562, 142–148. 10.1016/j.virol.2021.07.012. 34325286 PMC8299723

[ref39] Yang Q.; Hughes T. A.; Kelkar A.; Yu X.; Cheng K.; Park S.; Huang W.-C.; Lovell J. F.; Neelamegham S. Inhibition of SARS-CoV-2 viral entry upon blocking N- and O-glycan elaboration. eLife 2020, 9, e61552 10.7554/eLife.61552. 33103998 PMC7685702

[ref40] Tian C.; Kasavajhala K.; Belfon K. A. A.; Raguette L.; Huang H.; Migues A. N.; Bickel J.; Wang Y.; Pincay J.; Wu Q.; Simmerling C. ff19SB: Amino-Acid-Specific Protein Backbone Parameters Trained against Quantum Mechanics Energy Surfaces in Solution. J. Chem. Theory Comput. 2020, 16, 528–552. 10.1021/acs.jctc.9b00591. 31714766 PMC13071887

[ref41] Kirschner K. N.; Lins R. D.; Maass A.; Soares T. A. A Glycam-Based Force Field for Simulations of Lipopolysaccharide Membranes: Parametrization and Validation. J. Chem. Theory Comput. 2012, 8, 4719–4731. 10.1021/ct300534j. 26605626

[ref42] Izadi S.; Anandakrishnan R.; Onufriev A. V. Building Water Models: A Different Approach. J. Phys. Chem. Lett. 2014, 5, 3863–3871. 10.1021/jz501780a. 25400877 PMC4226301

[ref43] Tian C.; Kasavajhala K.; Belfon K. A. A.; Raguette L.; Huang H.; Migues A. N.; Bickel J.; Wang Y.; Pincay J.; Wu Q.; Simmerling C. ff19SB: Amino-Acid-Specific Protein Backbone Parameters Trained against Quantum Mechanics Energy Surfaces in Solution. J. Chem. Theory Comput. 2020, 16, 528–552. 10.1021/acs.jctc.9b00591. 31714766 PMC13071887

[ref44] Bussi G.; Donadio D.; Parrinello M. Canonical sampling through velocity rescaling. J. Chem. Phys. 2007, 126, 014101 10.1063/1.2408420. 17212484

[ref45] Parrinello M.; Rahman A. Polymorphic transitions in single crystals: A new molecular dynamics method. J. Appl. Phys. 1981, 52, 7182–7190. 10.1063/1.328693.

[ref46] Darden T.; Perera L.; Li L.; Pedersen L. New tricks for modelers from the crystallography toolkit: the particle mesh Ewald algorithm and its use in nucleic acid simulations. Structure 1999, 7, R55–R60. 10.1016/s0969-2126(99)80033-1. 10368306

[ref47] Li L.; Li C.; Sarkar S.; Zhang J.; Witham S.; Zhang Z.; Wang L.; Smith N.; Petukh M.; Alexov E. DelPhi: a comprehensive suite for DelPhi software and associated resources. BMC Biophys. 2012, 5, 9 10.1186/2046-1682-5-9. 22583952 PMC3463482

[ref48] Eisenhaber F.; Lijnzaad P.; Argos P.; Sander C.; Scharf M. The double cubic lattice method: Efficient approaches to numerical integration of surface area and volume and to dot surface contouring of molecular assemblies. J. Comput. Chem. 1995, 16, 273–284. 10.1002/jcc.540160303.

[ref49] Duan L.; Liu X.; Zhang J. Z. H. Interaction Entropy: A New Paradigm for Highly Efficient and Reliable Computation of Protein-Ligand Binding Free Energy. J. Am. Chem. Soc. 2016, 138, 5722–5728. 10.1021/jacs.6b02682. 27058988

[ref50] Williams A. H.; Zhan C.-G. Fast Prediction of Binding Affinities of the SARS-CoV-2 Spike Protein Mutant N501Y (UK Variant) with ACE2 and Miniprotein Drug Candidates. J. Phys. Chem. B 2021, 125, 4330–4336. 10.1021/acs.jpcb.1c00869. 33881861 PMC8084269

[ref51] Zhang Y.; He X.; Zhai J.; Ji B.; Man V. H.; Wang J. In silico binding profile characterization of SARS-CoV-2 spike protein and its mutants bound to human ACE2 receptor. Briefings Bioinf. 2021, 22, bbab188. 10.1093/bib/bbab188 PMC8194596 34013346

[ref52] Nissley D. A.; Vu Q. V.; Trovato F.; Ahmed N.; Jiang Y.; Li M. S.; O’Brien E. P. Electrostatic Interactions Govern Extreme Nascent Protein Ejection Times from Ribosomes and Can Delay Ribosome Recycling. J. Am. Chem. Soc. 2020, 142, 6103–6110. 10.1021/jacs.9b12264. 32138505 PMC7312765

[ref53] Nguyen H. L.; Lan P. D.; Thai N. Q.; Nissley D. A.; O’Brien E. P.; Li M. S. Does SARS-CoV-2 Bind to Human ACE2 More Strongly Than Does SARS-CoV?. J. Phys. Chem. B 2020, 124, 7336 10.1021/acs.jpcb.0c04511. 32790406 PMC7433338

[ref54] Allen J. D.; Watanabe Y.; Chawla H.; Newby M. L.; Crispin M. Subtle Influence of ACE2 Glycan Processing on SARS-CoV-2 Recognition. J. Mol. Biol. 2021, 433, 166762 10.1016/j.jmb.2020.166762. 33340519 PMC7744274

[ref55] Mehdipour A. R.; Hummer G. Dual nature of human ACE2 glycosylation in binding to SARS-CoV-2 spike. Proc. Natl. Acad. Sci. U.S.A. 2021, 118, e2100425118 10.1073/pnas.2100425118. 33903171 PMC8126795

[ref56] Barros E. P.; Casalino L.; Gaieb Z.; Dommer A. C.; Wang Y.; Fallon L.; Raguette L.; Belfon K.; Simmerling C.; Amaro R. E. The flexibility of ACE2 in the context of SARS-CoV-2 infection. Biophys. J. 2021, 120, 1072–1084. 10.1016/j.bpj.2020.10.036. 33189680 PMC7661960

[ref57] Gobeil S. M.; Henderson R.; Stalls V.; Janowska K.; Huang X.; May A.; Speakman M.; Beaudoin E.; Manne K.; Li D.; Parks R.; Barr M.; Deyton M.; Martin M.; Mansouri K.; Edwards R. J.; Sempowski G. D.; Saunders K. O.; Wiehe K.; Williams W.; Korber B.; Haynes B. F.; Acharya P. Structural diversity of the SARS-CoV-2 Omicron spike; bioRxiv, 2022. 10.1016/j.molcel.2022.03.028 PMC8947964 35447081

[ref58] McCallum M.; Czudnochowski N.; Rosen L. E.; Zepeda S. K.; Bowen J. E.; Walls A. C.; Hauser K.; Joshi A.; Stewart C.; Dillen J. R.; Powell A. E.; Croll T. I.; Nix J.; Virgin H. W.; Corti D.; Snell G.; Veesler D. Structural basis of SARS-CoV-2 Omicron immune evasion and receptor engagement. Science 2022, 375, 864–868. 10.1126/science.abn8652. 35076256 PMC9427005

[ref59] Mannar D.; Saville J. W.; Zhu X.; Srivastava S. S.; Berezuk A. M.; Tuttle K. S.; Marquez A. C.; Sekirov I.; Subramaniam S. SARS-CoV-2 Omicron variant: Antibody evasion and cryo-EM structure of spike protein-ACE2 complex. Science 2022, 375, 760–764. 10.1126/science.abn7760. 35050643 PMC9799367

[ref60] Kyte J.; Doolittle R. F. A simple method for displaying the hydropathic character of a protein. J. Mol. Biol. 1982, 157, 105–132. 10.1016/0022-2836(82)90515-0. 7108955

