# Supplementary material for: SARS-CoV-2 Omicron Variant Binds to Human Cells More Strongly than the Wild Type: Evidence from Molecular Dynamics Simulation
Source: J Phys Chem B. 2022 Jun 20;126(25):4669–78. doi: 10.1021/acs.jpcb.2c01048 (PMC9235043; doi:10.1021/acs.jpcb.2c01048)
Supplement: Supplementary file 1 — jp2c01048_si_001.pdf [file jp2c01048_si_001.pdf]

SARS-CoV-2 Omicron Variant Binds to Human Cells More Strongly than Wild Type: Evidence from Molecular Dynamics Simulation

Hoang Linh Nguyen[1,2,3], Nguyen Quoc Thai[1,4], Phuong H. Nguyen[5] and Mai Suan Li[6,*]

[1]Life Science Lab, Institute for Computational Science and Technology, Quang Trung Software City, Tan Chanh Hiep Ward, District 12, Ho Chi Minh City, Vietnam

[2]Ho Chi Minh City University of Technology (HCMUT), Ho Chi Minh City 700000, Vietnam

[3]Vietnam National University, Ho Chi Minh City 700000, Vietnam

[4]Dong Thap University, 783 Pham Huu Lau Street, Ward 6, Cao Lanh City, Dong Thap, Vietnam

[5]CNRS, Universit´e de Paris, UPR9080, Laboratoire de Biochimie Th´eorique, Paris,France ; Institut de Biologie Physico-Chimique, FondationEdmond de Rothschild, PSLResearch University, Paris, France

[6]Institute of Physics, Polish Academy of Sciences, al. Lotnikow 32/46, 02-668, Warsaw, Poland

*Email: masli@ifpan.edu.pl

SUPPORTING INFORMATION

Table S1: The contributions of non-bonded interaction energy (kcal/mol) between protein-glycan, protein-protein of the hACE2-RBD complexes.

Glycan model	Variant	Interaction	RBD protein - hACE2 protein	RBD glycan - hACE2 glycan	RBD protein - hACE2 glycan	RBD glycan - hACE2 protein	Total
Homogeneous glycan model	WT	Electrostatic	-794.49 ± 12.59	-12.36 ± 4.45	-53.54 ± 6.82	4.05 ± 0.67	-856.33 ± 24.53
		van der Waals	-99.28 ± 0.84	-9.98 ± 2.81	-42.88 ± 5.81	-0.04 ± 0.01	-152.18 ± 9.47
	Omicron	Electrostatic	-1452.81 ± 25.27	-12.48 ± 6.72	-184.88 ± 14.64	4.44 ± 1.11	-1645.73 ± 48.34
		van der Waals	-90.75 ± 3.88	-44.28 ± 4.27	-10.34 ± 2.94	-0.04 ± 0.01	-145.40 ± 11.10
Heterogeneous glycan model	WT	Electrostatic	-781.75 ± 14.05	-8.06 ± 4.59	-176.02 ± 17.19	1.55 ± 0.26	-964.27 ± 36.09
		van der Waals	-96.52 ± 3.28	-9.63 ± 5.19	-54.93 ± 4.03	-0.05 ± 0.01	-161.13 ± 12.51
	Omicron	Electrostatic	-1483.45 ± 22.79	-10.86 ± 4.06	-418.01 ± 26.72	2.95 ± 1.51	-1909.37 ± 55.08
		van der Waals	-91.66 ± 1.52	-14.73 ± 4.99	-47.28 ± 3.62	-0.04 ± 0.01	-153.71 ± 10.14

Table S2: The ratio (%) of SASA of glycan molecules binding to hACE2, RBD in relation to total SASA.

Glycan model	Variant	ACE2	RBD
Homogeneous	Wild type	10.77 ± 0.51	3.70 ± 0.30
	Omicron	19.74 ± 0.51	3.73 ± 0.23
Heterogeneous	Wild type	13.71 ± 0.73	3.65 ± 0.39
	Omicron	23.32 ± 0.72	3.18 ± 0.23

Table S3: Charge (measured in e) of ACE2, RBD, ACE2-glycans and RBD-glycans.

Glycan model	Variant	ACE2	RBD	ACE2-glycans	RBD-glycans
Homogeneous	Wild type	-27.6	+2.8	0.6	0.2
	Omicron	-27.6	+5.8	0.6	0.2
Heterogeneous	Wild type	-24.6	+2.8	-2.4	0.2
	Omicron	-24.6	+5.8	-2.4	0.2

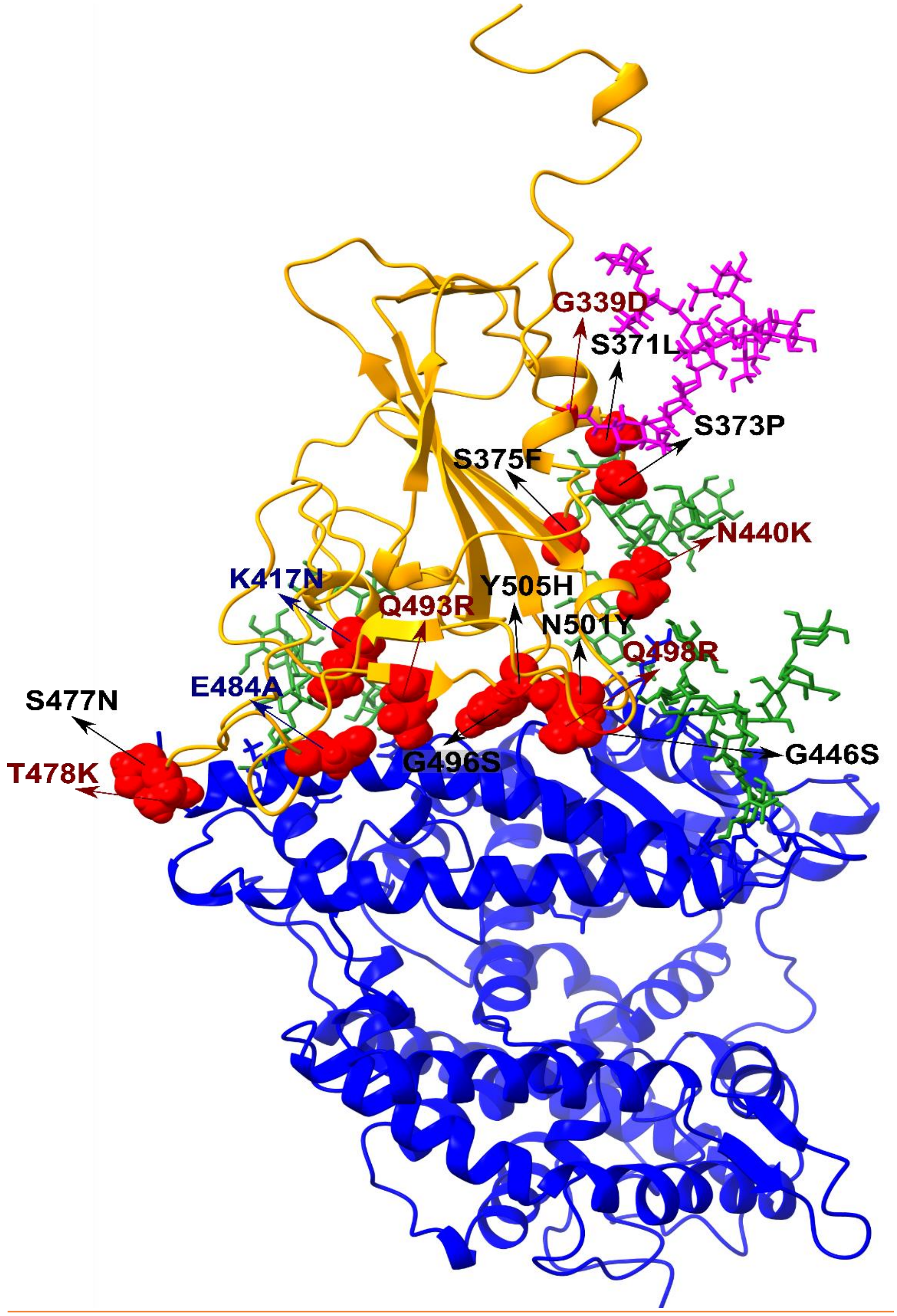

Figure S1: Shown are 15 mutations in the RBD of Omicron. The mutations that do not change charge have a black label. Mutations that are charged in Omicron, but neutral in WT have a maroon label. Mutations that are charged in WT, but neutral in Omicron have a navy label.

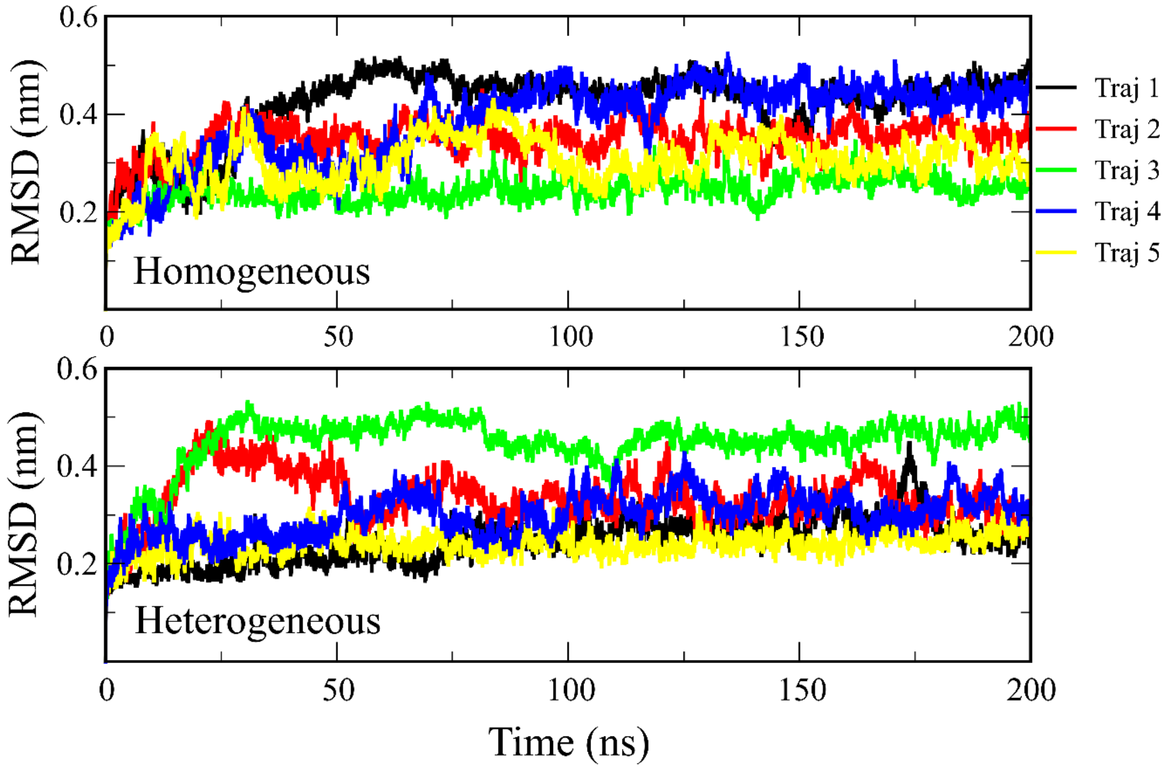

Figure S2: Time dependence of Cα RMSD of the WT RBD-hACE2 complex with the homogeneous (Upper) and heterogeneous (Bottom) models.

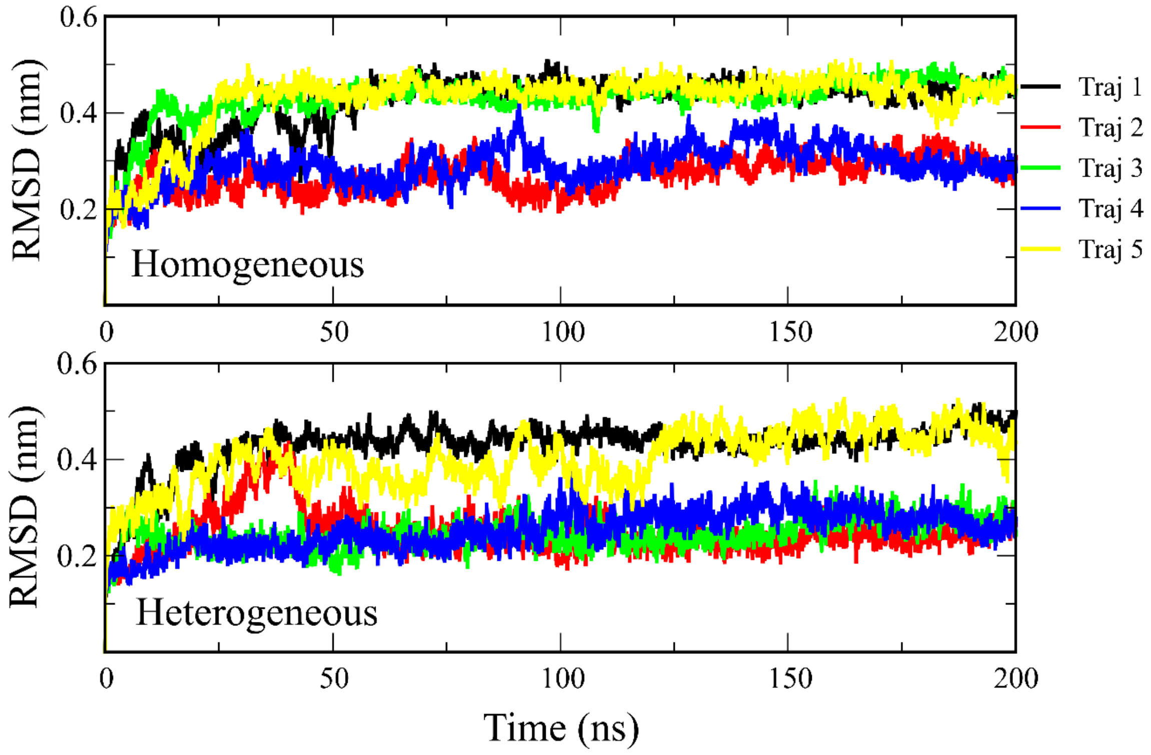

Figure S3: Time dependence of Cα RMSD of the Omicron RBD-hACE2 complex with homogeneous (upper) and heterogeneous (bottom) glycan models.

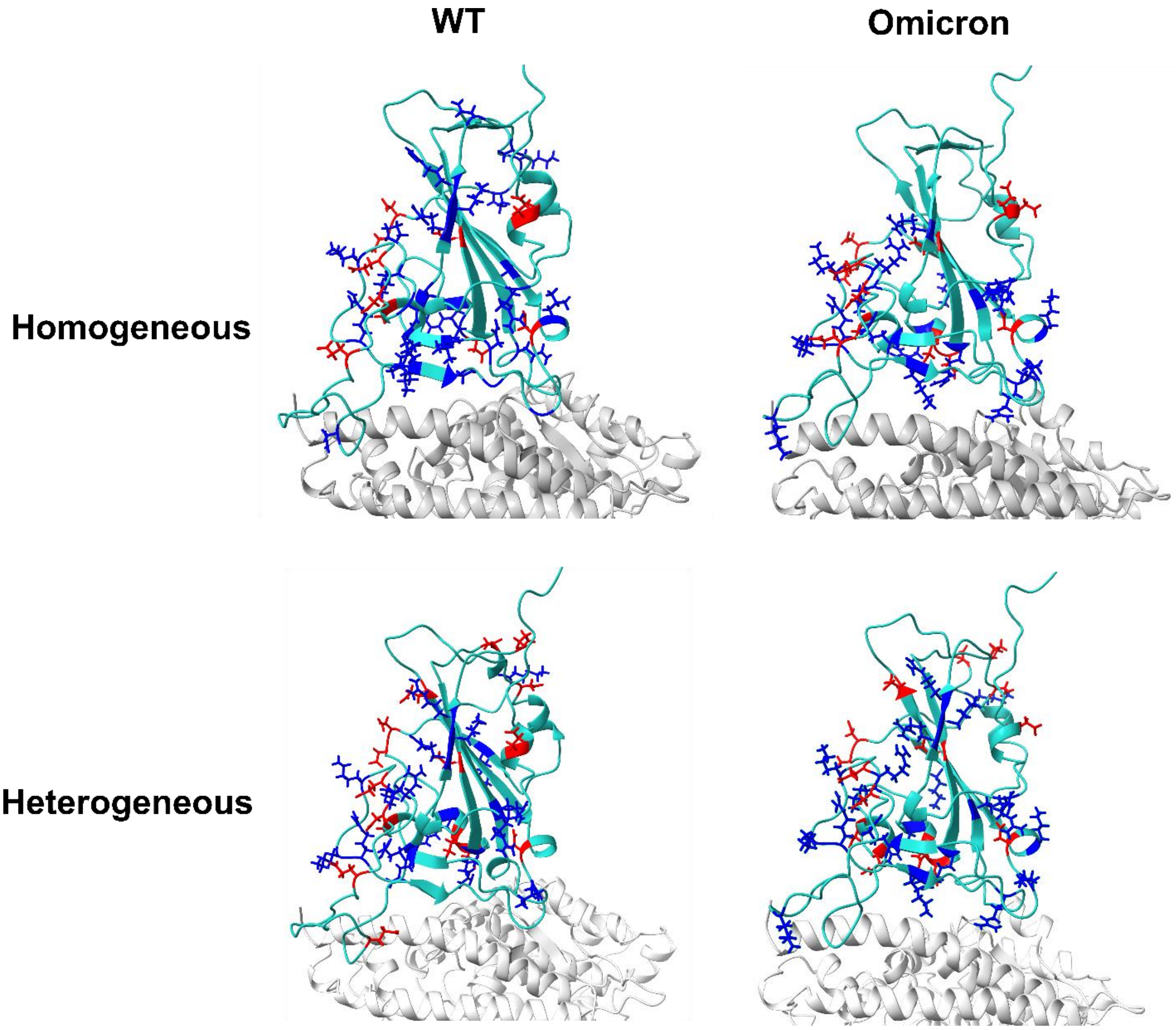

Figure S4: Residues of RBD that have an absolute interaction energy with hACE2 ≥ 150 kcal/mol. RBD is in cyan cartoon and hACE2 is in gray. Residues with an interaction energy ≤ -150 kcal/mol are in blue, while the residues with an interaction energy ≥ 150 kcal/mol are in red. For clarity glycans are not shown .

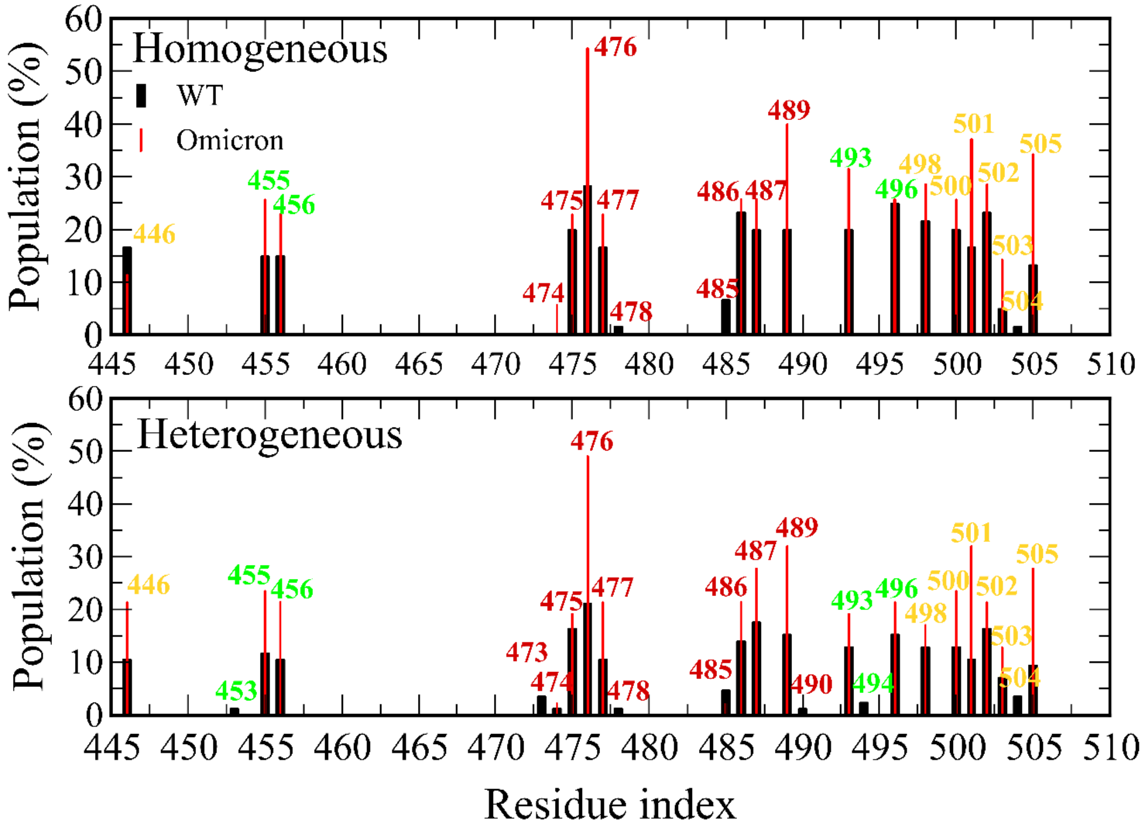

Figure S5: Population of side-chain contacts formed by RBD residues with hACE2 at the interface. The results were obtained using conformations sampled over the last 100 ns of MD simulation. The residues labeled in red, green and yellow colors correspond to the red, green and yellow regions, respectively.

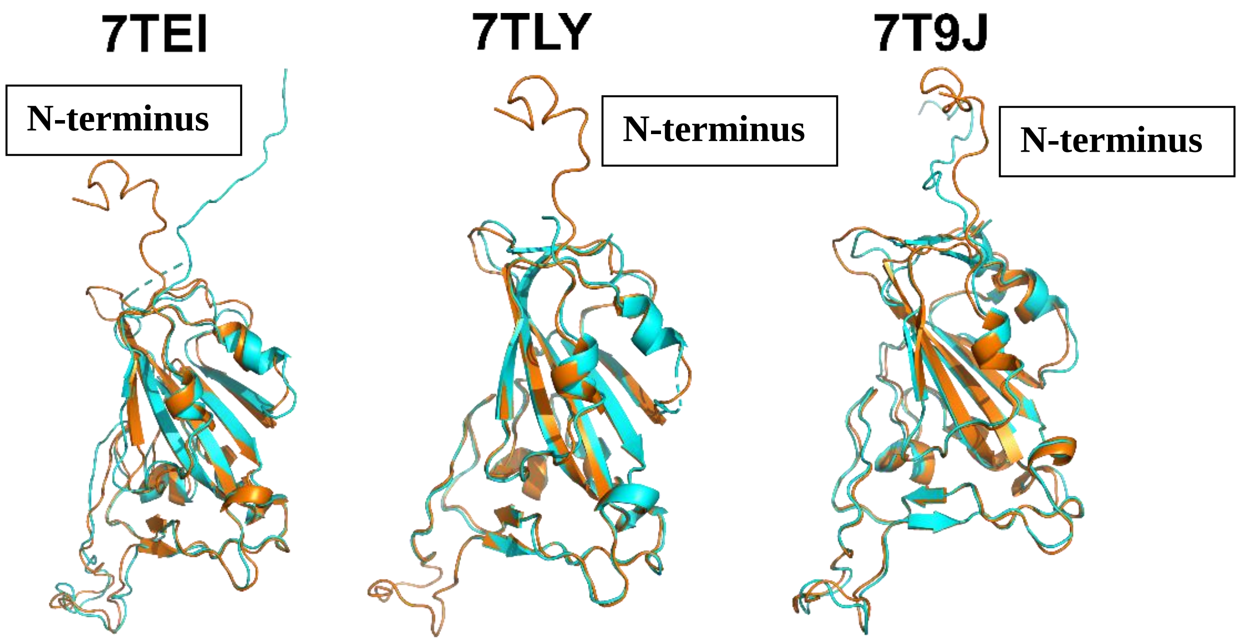

Figure S6: Our Omicron RBD (orange) is aligned with 3 RBD cryoEM structures from PDB (cyan). The main difference in RMSD between our model and cryoEM structures is related to the N-terminus.
